# A hepatic adrenal rest tumor with a benign gallbladder tumor: a case report and literature review

**DOI:** 10.3389/fonc.2025.1497448

**Published:** 2025-06-05

**Authors:** Yu Chen Wang, Jiang Li

**Affiliations:** ^1^ Shihezi University, The First Affiliated Hospital of Shihezi University, Shihezi, China; ^2^ First Affiliated Hospital, School of Medicine, Shihezi University, Shihezi, China

**Keywords:** immunohistochemical, case report, HART, HCC, benign gallbladder tumor

## Abstract

**Rationale:**

A hepatic adrenal rest tumor is an exceedingly rare disorder. Up to now, merely 13 cases of hepatic adrenal rest tumor have been documented in the English-language literature. Notably, the concurrent occurrence of a hepatic adrenal rest tumor and a benign gallbladder tumor in the same individual has not been previously reported. In this report, we present a case of a patient with a hepatic adrenal rest tumor who had a history of hepatitis B and was diagnosed with a benign gallbladder tumor. The tumors were successfully surgically resected, and the patient had a favorable prognosis during the follow-up period.

**Patient concerns:**

We report a 55-year-old male patient presenting with right upper abdominal discomfort, accompanied by chills and fever. He had a history of hepatitis B for more than 20 years but had not received regular medical evaluation and treatment. Serum tumor markers, including alpha-fetoprotein (AFP) and carcinoembryonic antigen (CEA), were within normal ranges. However, imaging studies showed a space-occupying lesion in the right posterior lobe of the liver and a metabolically active nodule detected by PET-CT at the base of the gallbladder.

**Diagnoses:**

Based on the patient’s past medical history and imaging findings, the preoperative diagnosis was hepatocellular carcinoma and gallbladder malignancy.

**Outcomes:**

The patient underwent atypical limited hepatectomy, radical cholecystectomy for gallbladder cancer, and resection of a right adrenal tumor.

**Conclusions:**

The histological report showed that the yellow soft tumors located in the right posterior lobe of the liver, adjacent to the adrenal region, were predominantly composed of clear cells. These cells were arranged in a pattern reminiscent of adrenal cortical tissue. Furthermore, immunohistochemistry showed that AFP (-), hepatocyte (-), melanoma antigen (MelanA) (+), inhibin-a (+), vimentin (+), synaptophysin (partially weak +), and chromogranin A (CgA) (-). The patient tolerated the surgery well, recovered uneventfully, and was discharged 11 days after surgery. There was no recurrence on follow-up.

## Introduction

Adrenal rest tumors are a rare group of tumors originating from the adrenal cortex, commonly found in the testes, spermatic cord, retroperitoneum, kidneys, and abdominal cavity (10), and have also been reported to occur in the intracranial, spinal, hepatobiliary, pancreas, lungs, and pericardial regions, but rarely in the adult liver. A search of the PubMed database using the term “hepatic adrenal rest tumor” was conducted, and a total of 15 results were retrieved. Among these, only 13 cases of hepatic adrenal rest tumors were identified. Two cases were reported to be associated with endocrine abnormalities and malignant histological features (10), while 11 cases were incidentally detected as non-functioning tumors. Among them, 12 cases were located in the posterior-superior subsegment of the right hepatic lobe, while the first report of a functionally active lesion in 1981 only indicated that it was located in the right hepatic lobe. A database search for “ectopic adrenal tissue in the liver” revealed that Vestfrid (1) was the first to report a case of ectopic adrenal tissue in the liver of a 3-day-old neonate, demonstrating that it consisted of adrenal cortical tissue of the fetal and adult types, and was the first case of ectopic adrenal tissue in the liver to be reported. We summarized all the retrieved cases and their characteristics in **Table 1**. Macroscopically, hepatic adrenal rest tumors (HARTs) typically present as well-demarcated, round, yellow nodular lesions. Histologically, HARTs share some similarities with hepatocellular carcinoma. The tumors are composed of round to polygonal cells. These cells are pale and lipid-rich, forming cell cords. These cell cords are separated by vascular channels or collagen bands. Additionally, the tumors contain hyaline or foamy cells, which are arranged in trabecular patterns. The imaging findings are usually characterized using MRI, with fat accumulation and hypervascularization as the two prominent features, but the latter is not specific and is often indistinguishable from hepatocellular carcinoma (HCC) or angiomyolipoma (AML). A hepatic adrenal rest tumor includes ectopic intrahepatic adrenal tissue and adrenal-hepatic fusion. Prolonged overstimulation of this tissue can lead to hyperplasia, sometimes even mimicking a true tumor. As reported by Yong et al. (15), in a case of intrahepatic adrenocortical adenoma resulting from adrenal fusion, it was difficult to distinguish this lesion from HCC because of the similar imaging features to those of HCC. Moreover, the yellowish incision surface of the tumor suggests the presence of fat components, which further emphasizes the necessity of differentiating between intrahepatic adrenocortical adenoma and HCC, as detailed in **Table 2**. The definitive diagnosis of hepatic adrenal rest tumors relies on the combined results of histology and immunohistochemistry. The lesion tissue consisted of many microvesicle-like cells arranged in a vesicular or trabecular pattern, similar to the characteristics of adrenal cortical tissue. In addition, immunohistochemical staining showed that tumor cells exhibited negative staining for alpha-fetoprotein (AFP), anti-endomysial antibody (EMA), and hepatocyte paraffin 1 (HepPar-1). They showed weakly positive staining for synaptophysin and negative staining for chromogranin A (CgA). Positive staining was observed for melanoma antigen (melan A), inhibin-a, and vimentin. CD34 was positive (vascular +), and the positive index of Ki-67 was approximately 4%.

**Table 1:** Clinical features of cases of hepatic adrenal rest tumor.

Author and year of publication	Age and sex	Hepatitis C virus	Functional status	Location	Tumor size (cm)	Malignant transformation
Vestfrid, 1980 (1)	3-day neonate	No	No	Liver parenchyma	0.2	No
Wallace et al., 1981 (2)	23, F	No	Yes	Right hepatic lobe	18*15*15	Yes
Altieri et al., 1985 (3)	21, F	No	Yes	Segment 7	10	Yes
Arai et al., 2000 (4)	62, M	Yes	No	Segment 7	2.2	No
Tajima et al., 2001 (5)	55, F	No	No	Segment 7	2.5	No
Baba et al., 2008 (6)	67, F	No	No	Segment 7	1.5	No
Soo et al., 2014 (7)	47, F	No	No	Segment 7	3.4	No
Ishida et al., 2014 (8)	81, F	No	No	Segment 7	3.3	No
Tajiri et al., 2015 (9)	50, F	No	No	Segment 7	3	No
Enjoji et al., 2017 (10)	67, F	No	No	Segment 7	1.4	No
Park et al., 2017 (11)	56, F	No	No	Segment 7	3.4	No
Park et al., 2017 (11)	64, F	No	No	Segment 6	2.2	No
Chen et al., 2021 (12)	44, M	Yes	No	Segment 6	7.8	No
Al-Taee et al., 2022 (13)	72, F	No	No	Segment 7	2.6	No
Zang et al., 2022 (14)	41, M	No	No	Segment 7	4	No

M male; F female.

**Table 2:** Comparison of characteristics of HART caused by two different growth patterns.

	Main features	Mechanism of occurrence	Results	Incidence
Hepatic adrenal fusion	Located between the right lobe of the liver and the right adrenal gland, without a fibrous septum. They are adherent and have irregular borders.	The fibrous envelope between the liver and adrenal glands is impaired, resulting in mixed growth of hepatic and adrenal parenchymal cells.	May develop into various benign and malignant tumors of the liver and adrenal glands	9.9%, positively correlated with age
Adrenal ectopic	The tumor that it develops into is located in the liver, with a fibrous septum between it and the adrenal glands, and does not adhere to them.	During development, the adrenal glands free themselves to continue to grow and function in other parts of the body.	Adrenal hyperplasia, adenoma, or cancer may occur.	Newborns or children 50%, adults 1%

In addition, the concurrent occurrence of a HART and a benign gallbladder tumor occurring in the same individual has never been previously reported. In this paper, we report a patient with a history of chronic hepatitis B who had a hepatic adrenal rest tumor combined with benign tumors of the gallbladder, who underwent surgical treatment and was followed up without recurrence or metastasis.

## Case report

A 55-year-old man presented with intermittent pain in his right upper quadrant, accompanied by chills and high fever with a temperature of up to 40°C. The admission CT examination revealed a space-occupying lesion in the S6 segment of the right lobe of the liver. Physical examination of the abdomen showed that it was soft and tender with pressure pain, predominantly in the right upper abdomen, with no rebound pain; the liver, spleen, and gallbladder were not palpable or enlarged. The patient had a 20-year history of chronic viral hepatitis B. Routine blood and biochemical tests showed no obvious abnormalities, with high leukocytes (9.7*10^9/L), decreased prealbumin (121 mg/L), decreased high-density lipoprotein (HDL) (0.81 mmol/l), and decreased creatine kinase (47 U/L). The anti-hepatitis A virus (HAV) IgM antibody test results were negative. As for the hepatitis B serological markers (commonly known as “hepatitis B two pairs and a half”), the hepatitis B e antibody (HBeAb), hepatitis B core antibody (HBcAb), and hepatitis B surface antigen (HBsAg) were positive and the anti-hepatitis C virus (HCV) antibody test results were negative. The levels of tumor markers, including AFP, carcinoembryonic antigen (CEA), carbohydrate antigen (CA) 12-5/19-9/72-4, and cytokeratin 19 (CK19), were all within the normal range. Therefore, gallbladder adenomyomatosis (GBA) with infection and hepatic mass lesions was initially considered.

**Figure 1:**
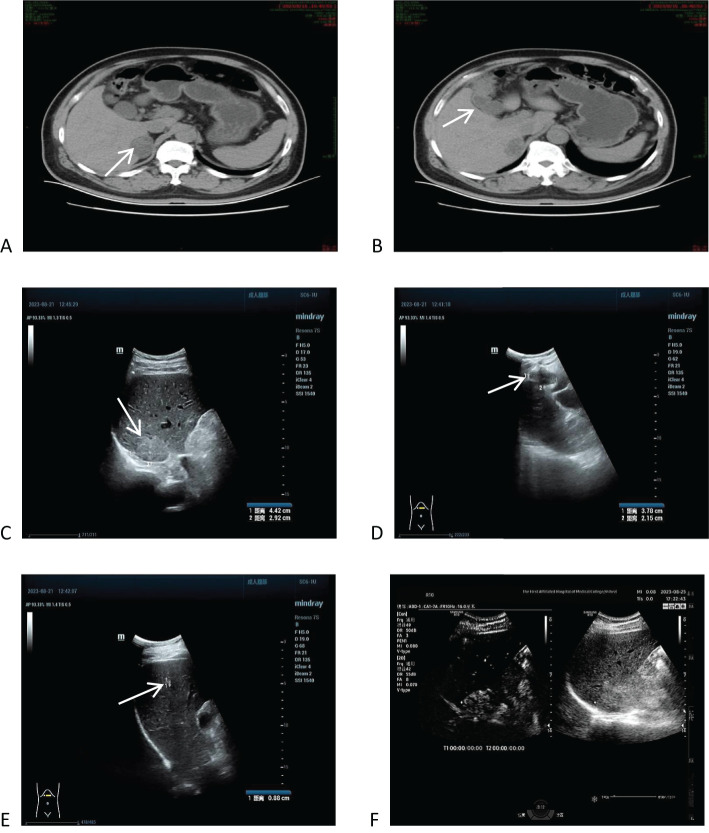
Computed tomography (CT) and ultrasound imaging of the upper abdomen, and ultrasonographic findings. **(A)** CT images showed a 3.8cm × 4.1cm × 2.9cm mass of hypodense opacity in the right adrenal region. **(B)** The bottom and wall of the gallbladder were obviously thickened and rough, with significant enhancement on the enhancement scan. **(C)** Ultrasound showed that there was a hypoechoic area with a size of about 4.4cm × 2.9 cm between the liver and the kidney. **(D)** One of the slightly elevated echoes was seen in the liver, which was 0.8cm × 0.7cm. **(E)** Ultrasonography shows a “fast in, fast out” pattern in the right lobe of the liver and a hypoechoic area at the gallbladder floor. **(F)** Further contrast-enhanced ultrasound showed high suspicion for hepatocellular carcinoma (HCC) and gallbladder carcinoma based on contrast agent dynamic changes.

**Figure 2:**
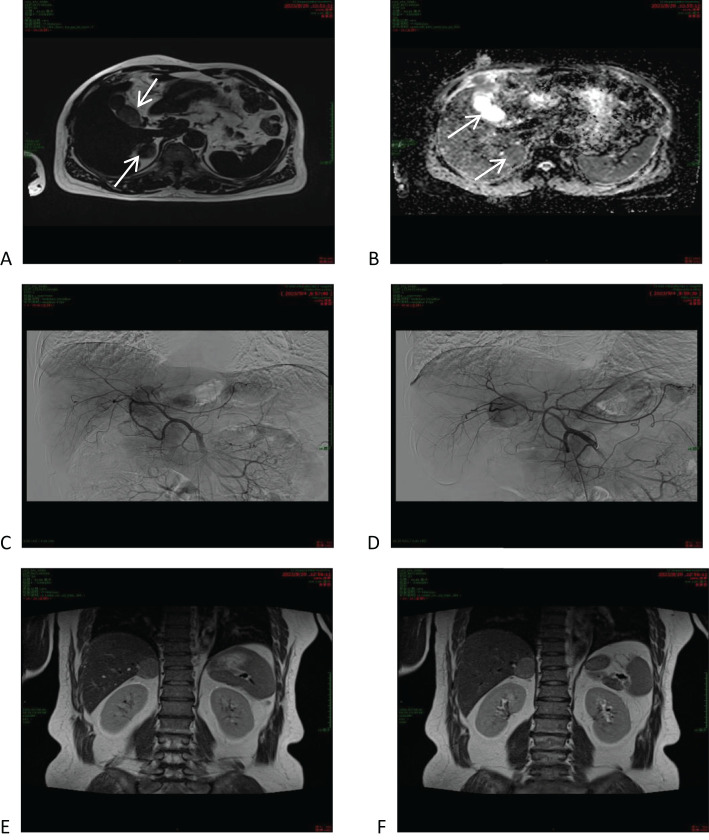
Magnetic resonance imaging (MRI) of the upper abdomen and hepatic arteriography. **(A, B)** MRI of the upper abdomen and hepatic arteriography showed that the right posterior lobe of the liver was slightly low-signal in T1WI and relatively high-signal in T2WI, and the lumen of the gallbladder was isosignal in T1WI and slightly high-signal in T2WI. **(C, D)** Hepatic arteriography shows irregularly disorganized tumor vessels proximal to the right hepatic artery. **(E, F)** CT showed that the mass was located in the lower part of the right posterior lobe of the liver, adjacent to the right adrenal gland.

**Figure 3:**
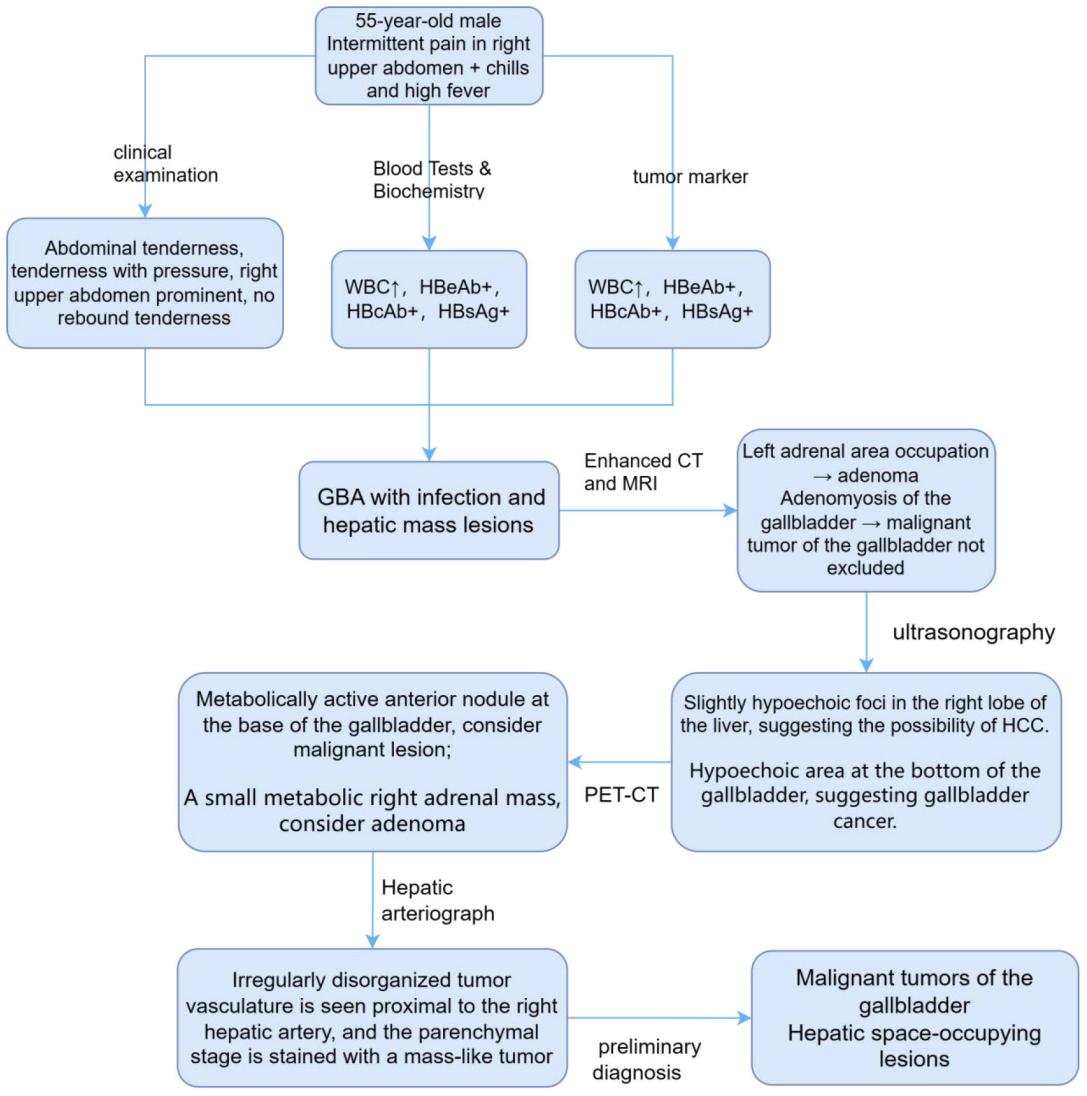
Diagnostic and treatment approach for this case.

In order to confirm the diagnosis, the following further imaging examinations were performed. ① The patient underwent dynamic contrast-enhanced computed tomography (CT) to evaluate the right hepatic mass. The CT images showed a 3.8cm × 4.1cm × 2.9cm mass of hypodense opacity in the right adrenal region, which was not clearly demarcated from the right posterior lobe of the liver, and the internal density was slightly uneven (**Figure 1A**). The bottom and wall of the gallbladder were obviously thickened and rough; the gallbladder partially protruded outward, which was markedly strengthened by enhancement, and a low-density cystic structure was seen in the gallbladder (**Figure 1B**). ② Abdominal ultrasound showed that there was a hypoechoic with a size of approximately 4.4cm × 2.9 cm between the liver and kidney, with a clear boundary and close relationship with the liver, and a short line of colorful blood flow signals was seen in it using color Doppler flow imaging (CDFI) (**Figure 1C**). The gallbladder was slightly abnormal, with an unevenly thickened wall of 0.6 cm at the thicker part, and an uneven hypoechoic area of about 3.8cm × 2.1 cm was seen at the bottom (**Figure 1D**). Several punctate hyperechoic and flaky hyperechoic areas were seen in the liver, with clear borders, one of which was slightly elevated, approximately 0.8cm × 0.7cm (**Figure 1E**). ③ Magnetic resonance imaging (MRI) of the upper abdomen showed that there was a patchy abnormal signal shadow in the right posterior lobe of the liver and near the adrenal region, with relatively low signal in T1-weighted imaging (T1WI) and slightly high signal in T2-weighted imaging (T2WI), and the size of the shadow was approximately 3.6cm × 2.9cm × 3.7cm (**Figures 2A, B**). The T1WI signal in the gallbladder cavity was elevated, the gallbladder floor and wall were thickened in the shape of a sac, the gallbladder wall was rough and protruding locally, and T2WI showed high intensity at the tumor, with equal intensity in T1WI and more uniform enhancement on an enhanced scan (**Figures 2A, B**). Hepatocellular carcinoma and cholecystadenomyosis were considered (but gallbladder malignant tumor was not excluded). ④ Therefore, further ultrasonography was performed, and the arterial phase of the slightly hypoechoic area in the right lobe of the liver was enhanced earlier than that of the surrounding liver tissues, with rapid hyperenhancement, and the portal and delayed phases were earlier than that of the surrounding liver tissues, with the contrast agent showing a “fast-in-fast-out” pattern, suggesting that the possibility of HCC was high (**Figure 1F**). The arterial phase of the hyperechoic area at the bottom of the gallbladder was slightly earlier than that of the surrounding liver tissue, and the portal and delayed phases were earlier than that of the surrounding liver tissue, suggesting the possibility of gallbladder cancer (**Figure 2D**). ⑤ To further clarify the treatment plan, a positron emission tomography (PET)-CT examination was performed, suggesting a small amount of metabolism of the right adrenal mass, which was considered an adenoma. The anterior nodule at the base of the gallbladder was metabolically active, which was considered a malignant lesion. ⑥Furthermore, hepatic arteriography showed irregular disordered tumor vascularity proximal to the right hepatic artery, with a mass-like tumor staining in the parenchymal stage (**Figures 2C, D**). In addition, although the mass was located in the liver, it was poorly demarcated from the right adrenal gland, so we demonstrated this relationship with some imaging pictures from another body position (**Figures 2E, F**). In order to clearly illustrate our approach to diagnosis and treatment, we have provided diagrams (**Figure 3**).

In summary, it was recommended that an atypical limited hepatectomy, a radical cholecystectomy for gallbladder cancer, and a right adrenal tumor resection be performed. During the operation, severe adhesions were found between the stomach, intestines, omentum, and the lower edge of the liver. After separating the adhesions, obvious edema of the gallbladder and invasion of the lateral abdominal wall were observed. The gallbladder and surrounding liver tissue were completely removed and sent for pathological examination. Further exploration of the right posterior lobe of the liver near the adrenal gland revealed a yellow, soft tumor measuring 4 cm × 3 cm, which was completely resected and sent for pathological examination. The surgery was successful, and the patient’s recovery went smoothly. The patient was discharged 11 days after surgery. The surgical specimens were collected, processed into routine paraffin sections, stained with hematoxylin-eosin (HE) in a routine manner, and examined immunohistochemically (IHC). No postoperative adjuvant therapy was administered. The patient was followed up for almost 7 months. Abdominal ultrasound review revealed an irregular strong echo approximately 1.6 cm in length and diameter in the upper part of the right posterior lobe of the liver, which was considered a postoperative change after cholecystectomy. No other obvious abnormalities were detected. The patient had a good postoperative prognosis, with no evidence of recurrence or metastasis.

### Pathological findings

**Figure 4:**
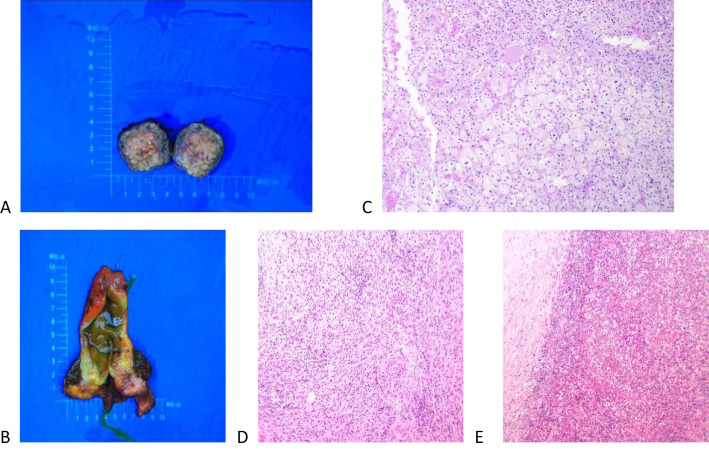
Macroscopic and microscopic findings of the excised tumor. **(A)** A yellow soft tumor in the right posterior lobe of the liver near the adrenal gland that was 3.5 cm × 2.5 cm × 1.6 cm in size and solid. **(B)** A grayish-white nodule, approximately 2.5 cm × 2.4 cm × 1.9 cm in size, was visible in a region of the gallbladder, with tissue adhesions. **(C)** Microscopically, the tumor consisted of clear cells arranged in a manner similar to adrenal cortical tissue, and the liver tissue surrounding the tumor showed early cirrhotic changes. **(D, E)** The gallbladder showed chronic cholecystitis, cholesterolosis, focal fibrotic nodules, and adenomyomatous nodule formation.

Macroscopically, there was a yellow soft tumor in the right posterior lobe of the liver near the adrenal region, closely associated with the liver, measuring 3.5 cm × 2.5 cm × 1.6 cm, with the tumor partially protruding from the surface of the liver (**Figure 4A**). A grayish-white nodule, approximately 2.5 cm × 2.4 cm × 1.9 cm in size, was visible in a region of the gallbladder, with tissue adhesions (**Figure 4B**). Microscopically, the tumor in the right posterior lobe of the liver was mainly composed of clear cells arranged in a manner similar to the characteristics of adrenocortical tissue. A few liver tissues around the tumor had mild edema of some hepatocytes, small bile ducts hyperplasia with mild dilatation in the confluent area, vitreous fibrous tissue hyperplasia with chronic inflammatory cell infiltration in some areas, and focal fibrous tissue segmentation encircling the hepatic lobules with a tendency of pseudofollicular formation, which was considered to be an indication of early cirrhosis (**Figure 4C**). The gallbladder showed chronic cholecystitis, cholesterolosis, focal fibrotic nodules, and adenomyomatous nodule formation (**Figures 4D, E**).

### Immunohistochemical studies

Immunohistochemical staining results showed that AFP was negative (-), EMA was negative (-), and hepatocytes were negative (-), which excluded a hepatocellular origin. Synaptophysin was weakly positive and chromogranin A was negative (CgA -), suggesting that the tumor cells had neuroendocrine features, as adrenocortical cells originated from the neuroendocrine system and stained positive for neuroendocrine markers. Melan A was positive (+), inhibin-αwas positive (+), and vimentin was positive (+), indicating an adrenocortical tumor. CD34 was positive (vascular +) and the Ki-67 positive index was approximately 4%. Therefore, the final diagnosis of hepatic adrenal rest tumor was made on the basis of the above findings.

## Discussion

Morgagni (16) first found yellowish nodules characteristic of adrenal tissue near the adrenal glands in 1740. About 150 years later, Marchand (17) found remnants of adrenal tissue in fetal and infant cadavers, thus began to speculate about the origin of ectopic adrenal tissue, which mainly includes the following two aspects. 1) Abnormalities during embryonic development: During embryonic development of the adrenal glands, the primitive adrenal glands may partially or wholly merge into neighboring organs such as the kidneys or the liver to form true ectasia. In addition, fragmentation of the adrenal tissue may result in its ectopia, termed accessory or quasi-ectopia. 2) Differentiation of the adrenal glands: The adrenal gland derives its cortex from the somatic mesenchymal epithelium and its medulla from chromaffin ectodermal cells of the neural groove. During development, multiple fragments of the primitive adrenal primordium or secondary segregation may divide to form accessory glands, which are usually composed of cortical material only. In conclusion, the origin of ectopic adrenal tissue is associated with abnormal development and differentiation of the adrenal gland during embryonic development.

**Figure 5:**
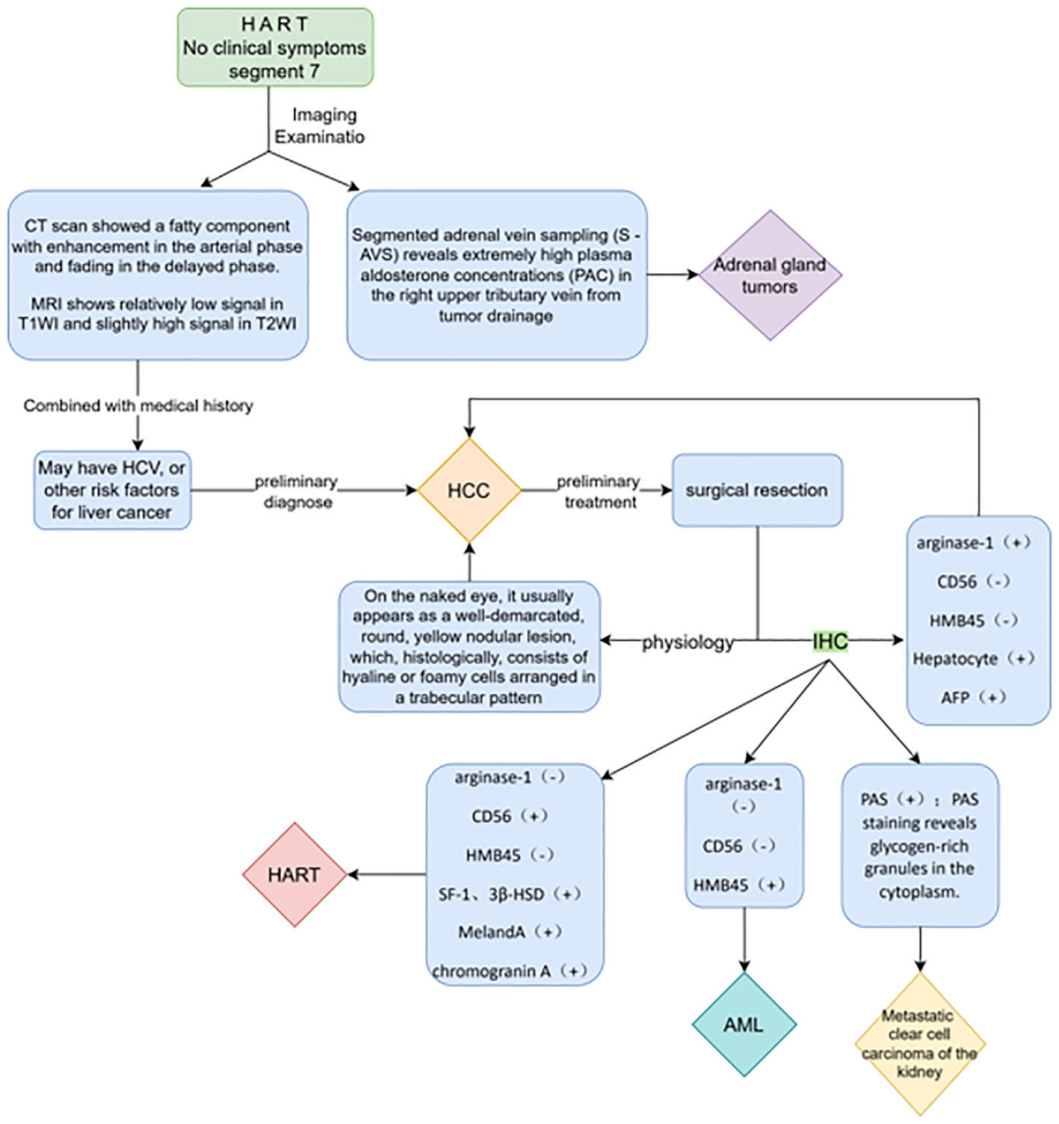
Differential diagnoses related to HART and disease characteristics.

The terms “ectopia” and “heterotopia” are used interchangeably, and ectopic tissues may also be called “accessory” or “rest” tissues (19). The mechanism of the HART reported in this paper may be the same. Adrenal ectopic tumors are very rare tumors that occur mainly in the testis, spermatic cord, retroperitoneum, kidney, and abdominal cavity, and less frequently in the liver. Adrenal ectopic tumors have received increasing attention since the first case of ectopic adrenal adenoma occurring in an inguinal hernia sac was reported by Morgagni (16) et al. in 1740. To date, 13 cases of HART have been reported in the English literature. The differential diagnoses includes HCC (lipid-rich or clear-cell HCC), AML, and metastatic renal clear-cell carcinoma (9). Special attention needs to be paid to differentiating HART from adrenal tumors (18) when it has a steroid hormone-secreting function (summarized in **Figure 5**).

The initial diagnosis of HART relies on imaging and pathologic evaluation, and since most are nonfunctioning benign tumors, there are often no typical clinical manifestations or symptoms. In this case, the patient presented with a combination of benign gallbladder tumors. The tumors caused discomfort, including intermittent right upper abdominal pain, leading to their detection. In fact, without IHC results, differentiating a HART from other tumors is often impossible due to the overlapping features with other malignant tumors. In particular, differentiating hepatic adrenal rest tumors from HCC before surgery is challenging, especially if the patient has a history of excessive alcohol consumption or other risk factors for HCC (e.g., history of hepatitis B). In this case, based on the patient’s history of hepatitis B, imaging manifestations, and the initial suspicion of gallbladder malignancy, we judged it to be more likely to be a gallbladder tumor combined with liver metastasis or a tumor of liver origin.

Reviewing the literature, Sugiyama et al. (9) proposed a diagnostic algorithm that includes CD56, arginase-1, and HMB45 (MART-1) immunostaining in the reported HART cases to prevent HART from being misdiagnosed as lipid-rich HCC. Therefore, we recommend that a biopsy should be performed first to further clarify the nature of the HART, taking into account the patient’s sex and age, and if the imaging shows that the mass is located in a favorable site (i.e., segment 7), presenting with an imaging manifestation similar to hepatocellular carcinoma but without specific clinical manifestations or past history, a needle biopsy should be performed first to further clarify the nature of the tumor. In addition, the specimen should be evaluated for the possibility of ectopic adrenal tissue to avoid unnecessary surgery.

Surgical resection is the preferred approach for hepatic adrenal rest tumors. A review of the existing literature on case reports reveals that patients who underwent surgical resection had favorable follow-up outcomes. Clinically, a definitive diagnosis is usually made only after surgical resection, based on further immunohistochemical results.

## Conclusion

In conclusion, we report a case of a patient with a history of hepatitis B who had a hepatic adrenal rest tumor (HART) combined with a benign gallbladder tumor. HART has been receiving increasing attention, and immunohistochemical analysis plays a crucial role in its diagnosis. Clinicians should be cautious when differentiating HART from hepatocellular carcinoma when mass lesions develop in patients with a history of hepatitis B or other risk factors. Complete resection remains the treatment of choice for HART, and its good prognosis is attributed to the benign nature of the tumor. A retrospective analysis of previously reported cases summarizes the most confusing and potentially overlooked differential diagnostic disorders and describes the pathogenesis of the tumor. A summary of the features of HART will help in early detection in future clinical practice.

## Data Availability

The original contributions presented in the study are included in the article/supplementary material. Further inquiries can be directed to the corresponding author.

## References

[1] Vestfrid MA . Ectopic adrenal cortex in neonatal liver. Histopathology. (1980) 4:669–72. doi: 10.1111/j.1365-2559.1980.tb02963.x 7439895

[2] Wallace EZ Leonidas JR Stanek AE Avramides A . Endocrine studies in a patient with functioning adrenal rest tumor of the liver. Am J Med. (1981) 70:1122–6. doi: 10.1016/0002-9343(81)90886-X 7234878

[3] Contreras P Altieri E Liberman C Gac A Rojas A Ibarra A. . Adrenal Rest Tumor of the Liver Causing Cushing's Syndrome: Treatment withKetoconazole preceding an Apparent Surgical Cure. J Clin Endocrinol Metab. (1985) 60:21–8. doi: 10.1210/jcem-60-1-21 3964792

[4] Arai K Muro H Suzuki M Oba N Ito K Sasano H. . Adrenal rest tumor of the liver: a case report with immunohistochemical investigation of steroidogenesis. Pathol Int. (2000) 50:244–8. doi: 10.1046/j.1440-1827.2000.01029.x 10792789

[5] Tajima T Funakoshi A Ikeda Y Hachitanda Y Yamaguchi M Yokota M. . Nonfunctioning adrenal rest tumor of the liver: radiologic appearance. J Comput assisted tomography. (2001) 25:98–101. doi: 10.1097/00004728-200101000-00018 11176302

[6] Baba Y Beppu T Imai K Masuda T Iyama K Sasano H. . A case of adrenal rest tumor of the liver: radiological imaging and immunohistochemical study of steroidogenic enzymes. Hepatol Res. (2008) 38:1154–8. doi: 10.1111/j.1872-034X.2008.00360.x 18564144

[7] Soo KL Azhar R Ooi LL . Hepatic adrenal rest tumour (HART): a case report. Ann Acad Med Singap. (2014) 43:120–2. doi: 10.47102/annals-acadmedsg. 24652433

[8] Ishida K Horii R Yamashita T Arai K Yamashita T Kagaya T. . Nihon shokakibyo gakkai zasshi. Journal of the Japanese Society of Gastroenterology. (2014) 111:2004–12. doi: 10.11405/nisshoshi.111.2004 25283230

[9] Sugiyama T Tajiri T Hiraiwa S Inomoto C Kajiwara H Kojima S. . Hepatic adrenal rest tumor: Diagnostic pitfall and proposed algorithms to prevent misdiagnosis as lipid-rich hepatocellular carcinoma. Pathol Int. (2015) 65:95–9. doi: 10.1111/pin.2015.65.issue-2 25572108

[10] Enjoji M Sanada K Seki R Ito T Maeda M . Adrenal rest tumor of the liver preoperatively diagnosed as hepatocellular carcinoma. Case Rep Surg. (2017) 2017:8231943. doi: 10.1155/2017/8231943 28706747 PMC5494576

[11] Park WY Seo HI Choi KU Kima A Kima YK Leea SJ. . Three cases of adrenocortical tumors mistaken for hepatocellular carcinomas/diagnostic pitfalls and differential diagnosis. Ann Diagn Pathol. (2017) 31:9–13. doi: 10.1016/j.anndiagpath.2017.05.016 29146062

[12] Chen J Wan X Lu Y Wang W Zhao D Lu Z. . An ectopic adrenocortical oncocytic adenoma in the liver highly mimicking hepatocellular carcinoma: case report and literature review. Diagn Pathol. (2021) 16:1–6. doi: 10.1186/s13000-021-01097-0 34218806 PMC8255004

[13] Al-Taee A Capenter D Garrett R Nazzal M Tetri B . Hepatic adrenal rest tumors mimicking hepatocellular carcinoma: A case report and review of the literature. Cureus. (2022) 14(11):e31343. doi: 10.7759/cureus.31343 36514652 PMC9741547

[14] Zang FL Yang B Zhang YH Yuhong G Yalei W Tingting D . Intrahepatic adrenocortical oncocytic adenoma arising from ectopic adrenal: report of a case. Zhonghua bing li xue za zhi= Chin J Pathol. (2022) 51:782–4. doi: 10.3760/cma.j.cn112151-20211203-00886 35922177

[15] Cho YS Kim JW Seon HJ Cho J-Y Park J-H Kim HJ. . Intrahepatic adrenocortical adenoma arising from adrenohepatic fusion mimicking hepatic Malignancy: Two case reports. Medicine. (2019) 98:e15901. doi: 10.1097/MD.0000000000015901 31169702 PMC6571242

[16] Morgagni GB . Cited by Wiesel, quod vidae. Postgrad Med J. (1980) 56(661):806–808. doi: 10.1136/pgmj.56.661.806 PMC2426068 7267489

[17] Schechter DC . Aberrant adrenal tissue. Ann Surg. (1968) 167:421. doi: 10.1097/00000658-196803000-00017 5638527 PMC1387073

[18] Baba S Miyoshi A Obara S Usubuchi H Terae S Sunahara M. . A case of Williams syndrome with suspected coexisting ectopic aldosterone-producing tumor in the liver. Endocrinol Diabetes Metab Case Rep. (2020) 2020. doi: 10.1530/EDM-20-0057 PMC7576637 33434178

[19] Tarçın G Ercan O . Emergence of ectopic adrenal tissues-what are the probable mechanisms? J Clin Res Pediatr Endocrinol. (2022) 14:258. doi: 10.4274/jcrpe.galenos.2021.2021.0148 34569220 PMC9422908

